# A Local Sensitivity Analysis of the Trial of Continuous or Interrupted Chest Compressions during Cardiopulmonary Resuscitation: Is a Local Protocol Change Required?

**DOI:** 10.7759/cureus.3386

**Published:** 2018-09-29

**Authors:** Brian Grunau, Joel Singer, Terry Lee, Frank X Scheuermeyer, Ron Straight, Robert Schlamp, Robert Wand, William F Dick, Helen Connolly, Sarah Pennington, Jim Christenson

**Affiliations:** 1 Emergency Medicine, St. Paul’s Hospital - University of British Columbia, Vancouver, CAN; 2 Epidemiology and Public Health, University of British Columbia, Vancouver, CAN; 3 Emergency Medicine, University of British Columbia, Vancouver, CAN; 4 Emergency Medicine, British Columbia Emergency Health Services, Vancouver, CAN; 5 Emergency Medicine, Providence Healthcare Research Institute, Vancouver, CAN

**Keywords:** cardiac arrest, cardiopulmonary resuscitation

## Abstract

Objective

The “Trial of Continuous (CCC) or Interrupted Chest Compressions (ICC) during Cardiopulmonary Resuscitation (CPR)” compared two CPR strategies for out-of-hospital cardiac arrest (OHCA). Although results were neutral, there was suggestion of benefit for ICC. However, nearly 50% of study patients had a protocol violation; regional variations may have played a role in protocol adherence and outcomes. We analyzed our British Colombia (BC) cohort to decide whether a protocol change from CCC to ICC was justified.

Methods

This was a post-hoc analysis of BC-enrolled study patients. The primary between-group comparison was favorable neurological outcome (modified Rankin scale ≤ 3) using intention-to-treat. Secondary analyses compared those treated per-protocol (adjusted) and the top compliant clusters (unadjusted). We classified protocol violations using a structured algorithm. We used logistic regression and computed the difference in probabilities using the marginal standardization method with bootstrapping to calculate confidence intervals.

Results

There were 3769 patients included, with a median age of 69 years (IQR: 56–80). There were protocol violations in 3.2% of those in the CCC group and 27% of those in the ICC group. In patients randomized to CCC or ICC, 11.2% and 10.8% (risk difference 0.42%; 95% CI -1.58, 2.41) had favorable neurological outcomes, respectively. In the per-protocol and top compliant clusters comparisons, risk differences were 0.25% (95% CI -1.70, 2.25) and 2.95% (95% CI -0.68, 6.58).

Conclusion

Our comparisons suggest that CCC may be the preferred strategy in our region and is likely not resulting in worse outcomes. Based on the original study and our local analysis, we found no compelling reasons to change our local strategy from CCC to ICC.

## Introduction

The “Trial of Continuous or Interrupted Chest Compressions during Cardiopulmonary Resuscitation” (“the CCC Trial”) was an unblinded multicenter cluster randomized trial of eight sites of the Resuscitation Outcomes Consortium (ROC) including 23,711 patients with out-of-hospital cardiac arrest (OHCA) [1]. The study compared two strategies for pre-intubation cardiopulmonary resuscitation (CPR): chest compressions interrupted for ventilations (ICC), and continuous chest compressions (CCC) uninterrupted for ventilations. This was the first clinical trial investigating differences in patient outcomes treated with these CPR strategies. As a primary outcome, the ICC group demonstrated a non-significantly higher survival rate (9.7% vs 9.0%, 95% CI on difference -1.5 to 0.1, p = 0.07). However, the tertiary outcome “hospital-free survival” (the number of days alive and permanently out of hospital during the first 30 days after the cardiac arrest) was significantly longer in the ICC arm and a per-protocol analysis demonstrated a significant benefit for ICC. Unfortunately a high proportion of protocol violations—nearly 50% among those for whom CPR process data was available [1]—makes the interpretation of this randomized trial difficult. An additional important consideration is the CPR strategy that local emergency medical systems (EMS) used prior to the study, as this may have had an impact on both the selection of patients for protocol violations and outcomes.

The British Columbia Ambulance Service (BCAS), which implemented CCC in 2006, enrolled approximately one-sixth of the CCC Trial patients. Although the trial did not demonstrate statistical superiority of ICC protocol, consideration of a local change to CCC was investigated, based on the “near significant” results of the primary outcome and superior results in secondary analyses. As this protocol change would involve significant investment, we sought to perform exploratory post-hoc analyses of the trial patients enrolled in British Columbia (B.C.), examining local protocol violations and outcomes within our EMS, with the pragmatic goal of determining whether a change in protocol was warranted. Although not sufficiently powered to demonstrate statistical differences, we hoped that these analyses would collectively assist with this decision.

## Materials and methods

The CCC trial methods

The methods of the CCC Trial (ClinicalTrials.gov Identifier: NCT01372748) have been previously described [2] and results reported [1]. Eight Resuscitation Outcomes Consortium sites, including 114 EMS agencies, performed this study of a cluster-randomized design with twice-yearly crossovers. Patients with non-traumatic OHCA who received EMS chest compressions were included; patients whose arrest was EMS-witnessed, those with a known advanced directive precluding resuscitation, those with an asphyxial or hemorrhagic arrest, those with a known pregnancy, prisoners, and those with a prior tracheostomy, were excluded.

The intervention was a CPR strategy of continuous chest compressions at 100 compressions per minute, with asynchronous positive-pressure ventilations at 10 per minute. In the control group CPR was interrupted every 30 compressions for two positive-pressure ventilations. Rhythm analyses were performed approximately every two minutes. After at least three cycles of compressions and rhythm analyses an advanced airway was inserted and all patients were treated with CCC.

Study setting

Subjects in the CCC trial were enrolled within the four urban regions of the province of B.C.: Victoria, Vancouver, the Fraser Valley, and Kelowna; a total of 3.3 million citizens (approximately 75% of BCs population) [3]. Enrollment was organized into 13 clusters. The Providence Health Care and University of British Columbia research ethics boards approved the study.

A coordinated EMS response from municipal fire departments (FD) and the single provincial BCAS provide pre-hospital emergency medical care for the province of B.C. FD responders are trained in basic cardiopulmonary life-support [4] including the use of automated external defibrillators (AED). Paramedics have either Advanced Life Support (ALS) [5] or Basic Life Support (BLS) [6] certification and work in pairs, typically with two paramedics of the same designation. All categories of responders are dispatched to an OHCA, typically arriving in the following order: FD, BLS, and ALS. BCAS policy indicates that all patients must undergo resuscitative efforts for at least 30 minutes unless contrary to family’s wishes or a “do not resuscitate” order is identified [7]. Transport of patients who do not achieve return of spontaneous circulation (ROSC) in the prehospital setting is uncommon [8].

The institutional ethical review boards of Providence Health Care and the University of British Columbia approved this study.

Data collection

All pre-hospital data, including time-stamped diagnostics, treatments administered, patient characteristics, and pre-hospital outcomes, were prospectively collected from standardized EMS template charting. We downloaded continuous electrocardiogram tracings with impedance determination of chest compressions from all EMS devices, and reviewed discharge outcomes for hospitalized patients via chart review.

This study was a post-hoc exploratory analysis of consecutive patients enrolled in the CCC Trial in B.C. [1]. All patients who had outcome data ascertained were included in this analysis.

Selection of participants and analysis groups

This study included three main comparisons. The primary analysis was an intention-to-treat (ITT) comparison of the outcomes of those randomized to CCC and ICC. As all patients in this comparison were included as randomized, we performed an unadjusted outcome comparison. A “Per Protocol” secondary analysis included only patients with protocol adherence, and compared the outcomes of those randomized to CCC to those randomized to ICC. As removing non-adherent patients could potentially cause significant differences in group characteristics, we performed an adjusted analysis for this comparison. Thirdly, we compared the outcomes of the top compliant enrolling clusters. The rationale for this comparison was these clusters best represented what was intended by the CCC randomized controlled trial, with high protocol compliance decreasing the potential for bias. We sequentially added clusters to this subgroup, in order of highest group compliance, until over 25% (determined a priori) of the total cohort were included. We employed an unadjusted outcome comparison, as patients were included as randomized. In addition to the three main analyses, additional crude or adjusted analyses (as applicable) were performed for all three comparisons.

Outcome measures and variable definitions

The primary endpoint was survival with favorable neurological outcome defined as a modified Rankin scale score ≤ 3 at hospital discharge as determined from the hospital discharge documents [9]. The secondary endpoint was survival to hospital discharge [9].

CPR-process data was acquired through monitor-defibrillators that identified each chest compression delivered. Research personnel, using a structured algorithm by manual review of the defibrillator download, classified protocol violations. Cases were considered ICC if 2/3 of the following criteria were met, with a pause defined as cessation of compressions for ≥ 3 seconds: (1) one or more pauses prior to first analysis; (2) two or more pauses, ≥25 compressions apart, during the first two-minute cycle (after the first analysis); (3) two or more pauses, ≥25 compressions apart, during the second two-minute cycle (after the second analysis).

The CCC Trial site principal investigator (Jim Christenson) examined all cases for which the treatment delivered was unclear to research personnel.

Data analysis

We used Microsoft Excel 2008 (Microsoft Corp, Redmond, WA, USA) and R version 3.2.4 (Foundation for Statistical Computing, Vienna, Austria) for analysis. Continuous variables are presented as means with standard deviations (if normally distributed) or medians with interquartile ranges.

For each comparison a crude and adjusted risk difference was calculated for the primary and secondary endpoint. Crude risk was compared between groups using Chi-square test and confidence interval of the risk difference was based on Wald asymptotic confidence limits. Logistic regression was used to assess the association between CPR strategy and clinical outcomes for the entire cohort and among the subgroups. An multivariate analysis was performed, adjusting for sex and covariates known to be associated with outcomes in OHCA patients: age, witnessed arrest, bystander CPR, interval from 911 call to EMS arrival, and initial cardiac rhythm [10-11]. Mixed effects regression was first used to assess the within cluster correlation; the estimated variance of the random effect from the model was zero and thus logistic regression without random effects was used instead. To estimate the risk difference between groups, we computed the adjusted difference in outcome probabilities using the marginal standardization method with bootstrapping to calculate confidence intervals.

## Results

Characteristics of study subjects

**Figure 1:**
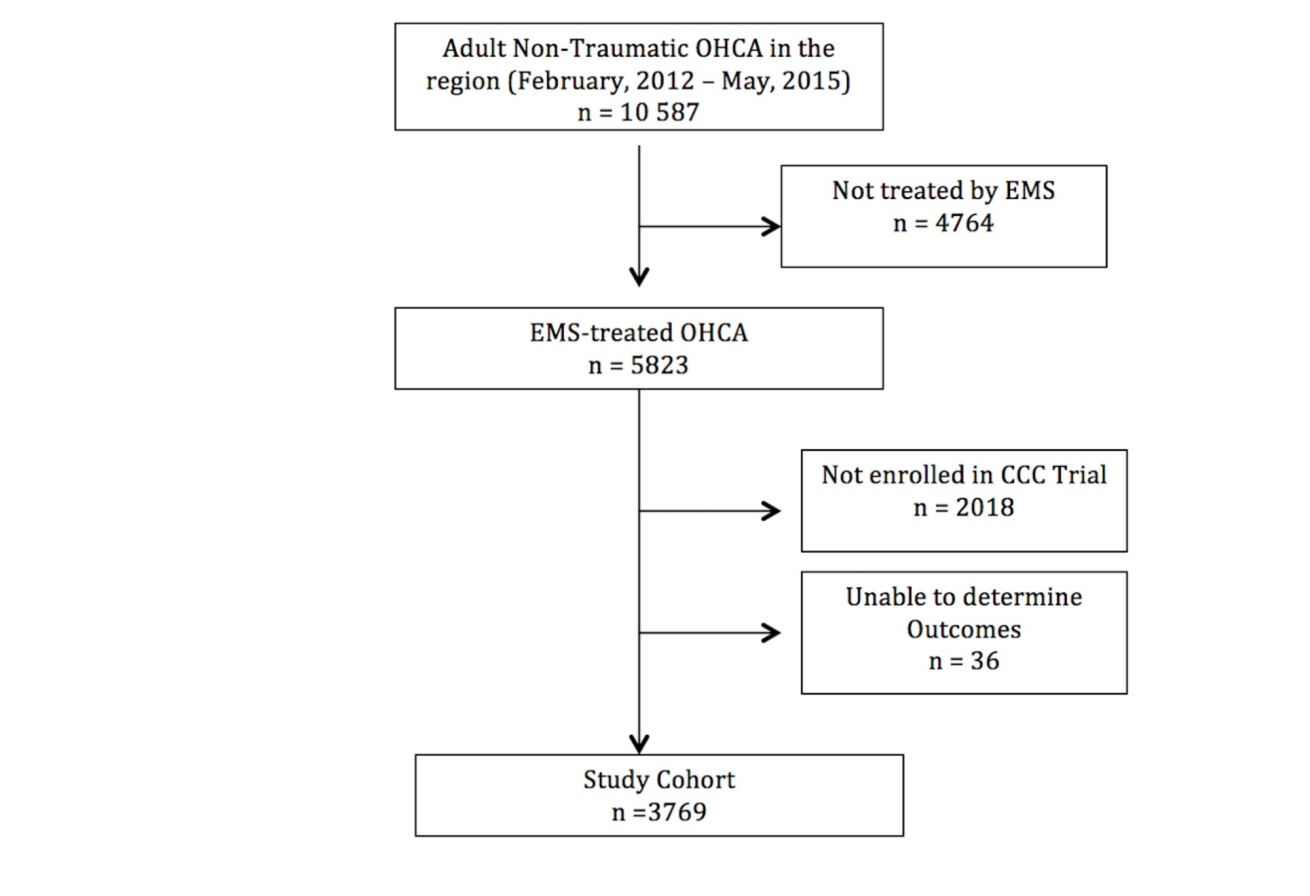
Procedure of composing study cohort. OHCA: Out-of-hospital cardiac arrest; EMS: Emergency medical service; CCC: Continuous chest compression.

During the period of enrollment in B.C. for the CCC Trial (February 2012 to May 2015) there were 5823 EMS-treated OHCAs, of whom 3769 were included in this analysis (Figure 1). The median age was 69 (IQR: 56–80), 44% were bystander witnessed, and 25% had initial shockable rhythms. Overall survival was 12.0%, with 11.0% of the cohort having favorable neurological outcomes. Patient characteristics of the three analyses of this study can be seen in Tables 1-3.

**Table 1:** Patient characteristics of intention to treat analysis. miss: Missing values; IQR: Interquartile range; CPR: Cardiopulmonary resuscitation; EMS: Emergency medical services; ROSC: Return of spontaneous circulation; ALS: Advanced life support.

	Continuous Chest Compressions			Interrupted Chest Compressions		
	n or median	% or IQR	miss	n or median	% or IQR	miss
Patients	1917			1852		
Age (years)	69 (57-80)	57-80	8	68	56-80	11
Male	1308	68.3%	3	1235	66.7%	1
Witnessed by bystander	838	43.7%	0	830	44.8%	0
Bystander CPR	1008	52.6%	0	956	51.6%	0
911 call to EMS arrival (min)	6.48	5.20-8.00	5	6.45	5.20-8.11	15
Initial shockable rhythm	508	26.5%	0	441	23.8%	0
ALS involvement	1848	96.4%	0	1780	96.1%	0
Chest compression fraction	0.88	0.83-0.91	377	0.80	0.74-0.86	400
Transported to hospital	918	47.9%	0	854	46.1%	0
ROSC	895	46.7%	0	827	44.7%	0

**Table 2:** Patient characteristics of per protocol analysis. CCC: Continuous chest compressions; ICC: Interrupted chest compressions; IQR: Interquartile range; miss: Missing value; CPR: Cardiopulmonary resuscitation; EMS: Emergency medical services; ALS: Advanced life support; ROSC: Return of spontaneous circulation.

	CCC Per Protocol			CCC Protocol Violations			ICC Per Protocol			ICC Protocol Violations		
	n or median	% or IQR	miss	n or median	% or IQR	miss	n or median	% or IQR	miss	n or median	% or IQR	miss
Number	1709			61			1183			499		
Age (years)	69	57-80	7	71	55-87	0	69	57-80	2	67	55-80	6
Male	1168	68.5%	3	39	63.9%	0	792	67.0%	1	339	67.9%	0
Witnessed by bystander	733	42.9%	0	30	49.2%	0	554	46.8%	0	208	41.7%	0
Bystander CPR	902	52.8%	0	32	52.5%	0	632	53.4%	0	240	48.1%	0
911 call - EMS arrival (min)	6.48	5.23-8.03	4	6.49	5.66-7.81	1	6.47	5.20- 8.10	7	6.42	5.30-8.19	4
Initial shockable rhythm	462	27.0%	0	14	23.0%	0	279	23.6%	0	129	25.9%	0
ALS involvement	1654	96.8%	0	56	91.8%	0	1147	97.0%	0	478	95.8%	0
Chest compression fraction	0.88	0.83-0.91	246	0.77	0.73-0.83	8	0.78	0.72-0.83	195	0.86	0.81-0.91	64
Transported to hospital	830	48.6%	0	26	42.6%	0	558	47.2%	0	219	43.9%	0
ROSC	807	47.2%	0	24	39.3%	0	535	45.2%	0	213	42.7%	0

**Table 3:** Patient characteristics of top compliant enrolling clusters analysis. miss: Missing value; IQR: Interquartile range; CPR: Cardiopulmonary resuscitation; EMS: Emergency medical services; ALS: Advanced life support; ROSC: Return of spontaneous circulation.

	Continuous Chest Compressions			Interrupted Chest Compressions		
	n or median	% or IQR	miss	n or median	% or IQR	miss
Number	592			471		
Age (years)	68	56-79	3	68	55-78	2
Male	405	68.5%	1	320	67.9%	0
Witnessed by bystander	263	44.4%	0	206	43.8%	0
Bystander CPR	332	56.1%	0	236	50.1%	0
911 call to EMS arrival (min)	6.72	5.40-8.08	2	6.10	5.41-7.97	1
Initial shockable rhythm	160	27.0%	0	103	21.9%	0
ALS involvement	559	94.4%	0	444	94.3%	0
Chest compression fraction	0.87	0.82-0.90	112	0.79	0.72-0.84	91
Transported to hospital	275	46.5%	0	203	43.1%	0
ROSC	267	45.1%	0	191	40.6%	0

Intention to treat analysis

**Figure 2:**
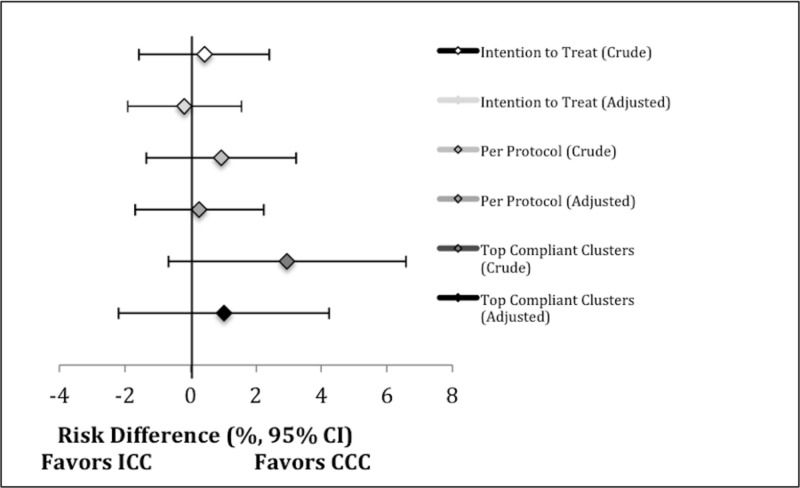
Crude and adjusted risk difference (with 95% CI) for favorable neurological outcome within each comparison. ICC: Interrupted chest compressions; CCC: Continuous chest compressions; CI: Confidence interval.

A total of 1917 patients were randomized to CCC and 1852 to ICC. Table 1 describes patient characteristics and outcomes of patients as randomized. Groups had similar baseline characteristics. In patients randomized to CCC and ICC, 12.2% and 11.8% survived to hospital discharge (absolute risk difference 0.38; 95% CI -1.69, 2.46) and 11.2% and 10.8% had favorable neurological outcomes (absolute risk difference 0.42; 95% CI -1.58, 2.41), respectively (Tables 4, 5; Figure 2). In the adjusted analysis of patients as randomized, the absolute risk difference for survival was -0.18 (95% CI -2.01, 1.66) and for neurological outcome was -0.19 (95% CI -1.92, 1.56), both in favor of ICC (Tables 4, 5; Figure 2).

**Table 4:** Unadjusted and adjusted survival for each analysis. CCC: Continuous chest compressions; ICC: Interrupted chest compressions.

	Unadjusted Survival			Adjusted
Analysis population	CCC	ICC	% Difference (95% CI)	% Difference (95% CI)
Intention to treat	233/1917 (12.2%)	218/1852 (11.8%)	0.38 (-1.69, 2.46)	-0.18 (-2.01, 1.66)
Per protocol	207/1709 (12.1%)	132/1183 (11.2%)	0.95 (-1.41, 3.32)	0.27 (-1.77, 2.36)
Top compliant clusters	74/592 (12.5%)	44/471 (9.3%)	3.16 (-0.58, 6.90)	1.21 (-2.24, 4.56)

**Table 5:** Unadjusted and adjusted neurological outcomes for each analysis. CCC: Continuous chest compressions; ICC: Interrupted chest compressions.

	Unadjusted Favorable Neurological Outcomes			Adjusted
Analysis population	CCC	ICC	% Difference (95% CI)	% Difference (95% CI)
Intention to treat	214/1917 (11.2%)	199/1852 (10.8%)	0.42 (-1.58, 2.41)	-0.19 (-1.92, 1.56)
Per protocol	191/1709 (11.2%)	121/1183 (10.2%)	0.95 (-1.34, 3.23)	0.25 (-1.70, 2.25)
Top compliant clusters	69/592 (11.7%)	41/471 (8.7%)	2.95 (-0.68, 6.58)	1.03 (-2.21, 4.23)

Per protocol analysis

Table 2 describes the patient characteristics of those who received the treatment as was randomized, and for those in whom the protocol was violated. Of those randomized to CCC and ICC respectively, there were 61 (3.2%) and 499 (26.9%) protocol violations; for 147 (7.7%) and 170 (9.2%) CPR process data was unavailable to determine if a violation occurred. In comparison to the per protocol ICC group, ICC protocol violations tended to have a lower proportion of witnessed arrests, bystander CPR, but a higher proportion of initial shockable rhythms.

Table 4 and Table 5 demonstrate the outcomes of the Per Protocol subgroup. The CCC group had a non-significant higher proportion of favorable neurological outcomes in the crude (risk difference 0.95; 95% CI -1.34, 3.23) and adjusted (risk difference 0.25; 95% CI -1.70, 2.25) analyses.

Top compliant enrolling clusters analysis

In the 13 enrolling clusters within B.C. the median group compliance by cluster was 83.7% (IQR: 80.4–84.7%). The top three clusters (representing 26% of the study population) had compliance rates of 93.2%, 85.4%, 84.7%; these groups enrolled 986 patients, of whom a total of 855 (86.7%) were treated as per protocol. Table 3 describes the characteristics of this subgroup. The CCC group tended to have a higher proportion of patients with bystander CPR and initial shockable rhythms.

Table 4 and Table 5 show the outcomes of this subgroup. The CCC group demonstrated a non-significant higher proportion of favorable neurological outcomes in the crude (risk difference 2.95; 95% CI -0.68, 6.58) and adjusted (risk difference 1.03; 95% CI -2.21, 4.23) analyses.

## Discussion

The CCC Trial was performed to determine the benefit of continuous chest compression CPR within the initial pre-intubation phase of resuscitation for OHCAs. Although neither strategy was proven to be superior, secondary results suggested benefit for ICC. In light of these results and the high proportion of protocol violations, we performed an exploratory sensitivity analysis of our site in order to inform the decision of whether a change in our chest compression policy was warranted. Our analyses do not demonstrate compelling evidence to warrant a change to an ICC strategy. Among EMSs who participated in the trial, the decision of which CPR strategy to use going forward has not been uniform, and systems—similar to ours—have had difficulty choosing the best option [12-14]. The data and analysis of this study may assist other regions in determining the best way forward for their individual systems.

Our study was an exploratory subgroup analysis of a high-quality effectiveness randomized controlled trial. Although the CCC trial did not disprove the null hypothesis and shows a statistically significant benefit of one CPR strategy, when looking beyond a binary p-value [15] the “near-significant” results of the study were suggestive of ICC benefit. Had the CCC trial primary analysis demonstrated ICC superiority, then a switch in our system would be pursued—however with the actual equivocal results we felt further local analyses were warranted, especially given the resource and opportunity cost of such a decision. Therefore, our purpose was not to contradict results of main study—this analysis lacked the required sample size to show statistically significant differences—but rather to answer two questions: (1) does local data suggest our current CCC protocol is resulting in harm to our patients? and, if not, (2) is it unlikely that our current strategy of CCC is worse than an ICC strategy?

Based on the inherent limitations in choosing appropriate non-inferiority margins [16], we did not design this pragmatic study as a non-inferiority study. However, our results suggest that our current protocol of CCC is likely not resulting in worse outcomes than an ICC strategy. Firstly, the three main comparisons—examining patients as randomized (ITT and Top Compliant Clusters) as well as the adjusted Per Protocol analysis—all demonstrate point estimates favoring CCC with the lower bound of the 95% CI for the risk difference greater than -2%. In our additional exploratory analyses, only the adjusted ITT point estimate favored ICC, and this difference was minimal. Secondly, the ITT analysis—with over one-quarter of the ICC group comprising of patients who were presumably treated with CCC—demonstrated the smallest difference between groups. If CCC does in fact result in better outcomes than ICC, then an ITT analysis that includes CCC-treated patients in the ICC arm would reduce any apparent superiority of the CCC arm. In the Per Protocol and Top Compliant Clusters Analyses, which reduced this cross-contamination, the differences between groups increased (favoring CCC). This was observed even after adjusting for baseline characteristics to mitigate bias, which may have been introduced with selectively chosen protocol violations.

A large proportion of protocol violations occurred in the CCC trial (45% protocol violations among the 18,571 randomized patients [78%] who had CPR process data available) with considerable variation in adherence between sites [1]. This may limit the benefits of the randomized control trial design (which assumes an equal proportion of unmeasured confounders between groups) and makes conclusions of this trial more difficult to interpret. Trial protocol adherence can be a significant obstacle, especially in open-label pre-hospital cardiac arrest studies [17], possibly as paramedics are confronted with the ethical dilemma of treating critically ill patients as per group assignment even if it contradicts their convictions of the best available treatment. While treatment site was not found to be a statistically significant effect modifier in the CCC trial (although this analysis may have been underpowered) it is unclear to what extent previous CPR strategy influenced protocol adherence at each site. BCAS and municipal BC FDs have utilized CCC as the standard form of chest compressions since 2006 and were taught that the highest possible chest compression fraction gave the best chances of survival; this metric was continuously monitored through defibrillator downloads. From our results it is clear that non-compliance with the protocol occurred much more frequently with the ICC group (27%) than with the CCC group (3%). Despite a great deal of paramedic education in protocol adherence and the rationale for the trial, we postulate that this imbalance may be due to both a belief that CCC is a better form of CPR, and that it may be easier to perform in the chaotic circumstances of a cardiac arrest. When examining our unadjusted comparisons all favored CCC, however these results were attenuated with adjustment for baseline characteristics, suggesting that—of the patients randomized to ICC—those with better prognostic factors were more likely to receive the CCC treatment than those with poor prognostic factors. This highlights the importance of our further analyses to explore outcome differences in subgroups with less contamination in order to reduce bias.

In view of the equivocal results of the multicenter CCC trial each EMS agency may be obligated to review its chest compression strategy, and evaluate the overall evidence to make a recommendation. It may be too simplistic to apply the aggregate results of a multicenter trial, and it is important to view the larger results within a local context. Although the best available evidence may be inconclusive with results that do not achieve statistical significance, leaders of medical organizations do not have the option of stating “there is insufficient evidence to make a recommendation”. Our conclusion, based on multiple local comparisons and the CCC Trial results, is that there is insufficient evidence to recommend a local change in CPR policy.

Limitations

This analysis was a single-site subgroup post-hoc analysis of a large randomized controlled trial that was not designed for this purpose, nor was the sample size sufficient to show significant differences. Regional variability within a large multicenter trial is to be expected, especially in studies with neutral results in which half of the sites may favor a different therapy. However, recent evidence indicates that there may be substantial geographic variations in the results of randomized trials; this may not simply be due to chance, but due to differences in local patients characteristics, local institutional resources, or concomitant treatments [18]. The external validity of this single-site analysis, where one approach was historically ingrained in care providers, is limited. Our method for determining protocol adherence may have misclassified some patients; in particular, the algorithm assumed ventilations were being performed during pauses in CPR without confirmatory data. Our algorithm classified the CPR pattern as ICC if as few as two pauses occurred per cycle for as few as three seconds, which may have erroneously coded CCC-provided patients as ICC; however, the small proportion of CCC protocol violations argues against this. The study intervention consisted of two slightly different CPR strategies during the first six minutes of cardiac arrest resuscitation. Measurable and unmeasurable characteristics and care processes of cardiac arrest other than those identified and adjusted for in this study—including post-resuscitation care—likely influenced outcomes. However, data acquired as part of the CCC Trial was of the highest quality to date, and provided the best possible answer to the question of CPR strategy.

## Conclusions

We performed an exploratory single-site sensitivity analysis from patients enrolled in the CCC Trial to assist in deciding whether a protocol change was warranted in our region. We found no compelling reasons to alter our current CPR policy from continuous chest compressions to interrupted chest compressions for ventilation. Our comparisons suggest that CCC may be the preferred strategy in our region and is likely not resulting in worse outcomes.

## References

[1] Nichol G, Leroux B, Wang H (2015). Trial of continuous or interrupted chest compressions during CPR. N Engl J Med.

[2] Brown SP, Wang H, Aufderheide TP (2015). A randomized trial of continuous versus interrupted chest compressions in out-of-hospital cardiac arrest: rationale for and design of the Resuscitation Outcomes Consortium Continuous Chest Compressions Trial. Am Heart J.

[3] (2018). Focus on geography series, 2011 census. Focus on Geography Series, 2011 Census.

[4] Berg RA, Hemphill R, Abella BS (2010). Part 5: adult basic life support: 2010 American Heart Association Guidelines for cardiopulmonary resuscitation and emergency cardiovascular care. Circulation.

[5] Link MS, Berkow LC, Kudenchuk PJ (2015). Part 7: adult advanced cardiovascular life support: 2015 American Heart Association Guidelines update for cardiopulmonary resuscitation and emergency cardiovascular care. Circulation.

[6] Kleinman ME, Brennan EE, Goldberger ZD (2015). Part 5: adult basic life support and cardiopulmonary resuscitation quality: 2015 American Heart Association Guidelines update for cardiopulmonary resuscitation and emergency cardiovascular care. Circulation.

[7] (2010). Resuscitation of Critically Ill Patients. Service Policy Number 6.4.13, Volume 2.

[8] Grunau BE, Reynolds JC, Scheuermeyer FX (2016). Comparing the prognosis of those with initial shockable and non-shockable rhythms with increasing durations of CPR: informing minimum durations of resuscitation. Resuscitation.

[9] Perkins GD, Jacobs IG, Nadkarni VM (2015). Cardiac arrest and cardiopulmonary resuscitation outcome reports: update of the Utstein resuscitation registry templates for out-of-hospital cardiac arrest: a statement for healthcare professionals from a task force of the international liaison committee on resuscitation. Resuscitation.

[10] Reynolds JC, Frisch A, Rittenberger JC, Callaway CW (2013). Duration of resuscitation efforts and functional outcome after out-of-hospital cardiac arrest: when should we change to novel therapies?. Circulation.

[11] Stiell IG, Wells GA, DeMaio VJ (1999). Modifiable factors associated with improved cardiac arrest survival in a multicenter basic life support/defibrillation system: OPALS Study Phase I results. Ontario Prehospital Advanced Life Support. Ann Emerg Med.

[12] (2017). 2016 Annual Report to the King County Council. http://www.kingcounty.gov/depts/health/~/media/depts/health/emergency-medical-services/documents/reports/2016-Annual-Report.ashx.

[13] Verbeek R. (2017). CCC trial demonstrated that continuous chest compressions and 30:2 CPR are equivalent. http://www.prehospitalmedicine.ca/wp-content/uploads/message/TO_2016Jan06_CCC-Trial_Implications.pdf.

[14] Uukkivi T (2017). Continuous chest compressions. http://www.lhsc.on.ca/About_Us/Base_Hospital_Program/Continuouschestcompressions-Memorandum.pdf.

[15] Wasserstein RL, Lazar NA (2016). The ASA’s statement on p-values: context, process, and purpose. Am Stat.

[16] Wangge G, Roes K, de Boer A, Hoes A, Knol M (2013). The challenges of determining noninferiority margins: a case study of noninferiority randomized controlled trials of novel oral anticoagulants. CMAJ.

[17] Jacobs IG, Finn JC, Jelinek GA, Oxer HF, Thompson PL (2011). Effect of adrenaline on survival in out-of-hospital cardiac arrest: a randomised double-blind placebo-controlled trial. Resuscitation.

[18] Yusuf S, Wittes J (2016). Interpreting geographic variations in results of randomized, controlled trials. N Engl J Med.

